# Molecular basis of differential nitrogen use efficiencies and nitrogen source preferences in contrasting Arabidopsis accessions

**DOI:** 10.1038/s41598-018-21684-4

**Published:** 2018-02-20

**Authors:** Jochen Menz, Tim Range, Johannes Trini, Uwe Ludewig, Benjamin Neuhäuser

**Affiliations:** Institute of Crop Science, Nutritional Crop Physiology, University of Hohenheim, Fruwirthstr. 20, 70593 Stuttgart, Germany

## Abstract

Natural accessions of *Arabidopsis thaliana* differ in their growth and development, but also vary dramatically in their nitrogen use efficiencies (NUE). The molecular basis for these differences has not been addressed yet. Experiments with five contrasting accessions grown in hydroponics at different levels of inorganic nitrogen confirmed low NUE of Col-0 and higher NUE in Tsu-0. At constant external nitrogen supply, higher NUE was based on nitrogen capture, rather than utilization of nitrogen for shoot biomass. This changed when a limited nitrogen amount was supplied. Nevertheless, the total NUE sequence remained similar. Interestingly, the two most contrasting accessions, Col-0 and Tsu-0, differed in the capture of single inorganic nitrogen sources, reflected by the differential consumption of ^15^N label from ammonium or nitrate, when supplied together. Tsu-0 acquired more nitrate than Col-0, both in roots and shoots. This preference was directly correlated with the expression of certain nitrogen uptake and assimilation systems in the root. However, early transcriptional responses of the root to nitrate deprivation were similar in both accessions, suggesting that the sensing of the external lack of nitrate was not different in the more nitrogen use efficient accession. Thus, a robust rapid nitrate-deprivation signaling exists in both genotypes.

## Introduction

Nitrogen use efficiency is a crucial trait for environmental competitiveness within mixed plant communities and in intensive crop production. In modern agroecosystems, balancing the high nitrogen (N) inputs with high quality yields is a demanding challenge. For decades, the nitrogen use efficiency (NUE) of modern cereal crop farming systems has been rather low, globally only at around 30%^1^. The global nitrogen usage by plants in farming systems remains low, although some countries improved their nitrogen usage by better fertilizer management, which is of most critical importance for reducing N inputs^2^. Current estimates suggest that even under ideal high yielding conditions in Europe, the NUE of realistic agrosystems is below 75%. This is mainly due to unavoidable nitrogen depositions in soils, gaseous losses and nitrate leaching^3^. Apart from proper field management, sustaining crop farming may therefore also require the genetic improvement of plants.

Over the years, the nitrogen use efficiency (NUE) of plants has been defined in many different ways. These are generally reflecting the focus on agronomic, ecological or breeding issues. Typically, the NUE definitions relate the shoot biomass or grain generated to the nitrogen taken up or the nitrogen which was available to the plant^4,5^. Unfortunately, genetic researchers frequently define the NUE different than agronomists and therefore they seldom address an agronomically relevant NUE in their studies^6^. However, genetic work identified an increasing number of genes, such as glutamine synthetase, to be crucial for superior nitrogen use efficiency. In transgenic plants, genetically improved nitrogen usage of crops and *Arabidopsis* was obtained^5,7^. More recently, rice varieties were genetically improved by the altered expression of various nitrate transporter genes^8,9^, pointing to the uptake of nitrate as crucial factor for the NUE in these varieties.

For the comparison of the physiologic plant performance of certain genotypes at different nitrogen supply levels, NUE is frequently dissected into easily accessible components, such as the capture of nitrogen by the roots (=N uptake efficiency, NUpE), as well as the biomass (or grains) produced per nitrogen acquired into the shoot (=N utilization efficiency, NUtE)^10^. The product of NUpE and NUtE is NUE, but as the biomass strongly depends on the nitrogen taken up, uptake and utilization efficiency are not independent of each other. By definition, the nitrogen use efficiency increases at lower N supply. Species utilizing C4 metabolism generally exhibit a superior nitrogen usage than C3 plants, as C4 plants reduce photorespiration and need far less RUBISCO, the major protein responsible for up to 50% of the N-content in leaves^11^. The concept of NUE is also useful for genotype and mutant comparisons within a species^4,5^. Besides rice as a model plant for cereals, *Arabidopsis thaliana* remains the model dicot plant. However, the *Arabidopsis* accession Col-0, for which most genetic resources are available (e.g. T-DNA insertion lines of candidate genes) and most genetic experiments have been performed, displays low intrinsic NUE, when compared to other accessions^12^. In a specific set of accessions, substantial natural variation was encountered in the uptake efficiency (NO_3_^−^ taken up per available NO_3_^−^) and utilization efficiency (shoot biomass per NO_3_^−^ taken up). When grown in sand-based culture supplemented with nitrate, five NUE classes within a population of 21 *Arabidopsis* accessions were identified, with Col-0 being least efficient and little responding to higher nitrate^12–14^. In another study with accessions grown on agar plates, Col-0 was again little responding to higher nitrate^15^. In all these studies, nitrate was used as exclusive N-source for determining NUE, although in native soils, ammonium is additionally available and is even the preferred N source of *Arabidopsis* in short-term uptake experiments^16^. While the short-term root uptake of ammonium or nitrate strongly varies among different accessions, it is less clear whether this translates into differential long-term assimilation, i.e. differences in assimilated N from each N-form.

Here, we therefore re-addressed the total nitrogen use efficiency of a subset of five contrasting *Arabidopsis* accessions. We hypothesized that when ammonium and nitrate are both available to the plants, the relative performance and NUE of the genotypes might differ from that with nitrate as the sole nitrogen source. Contrasting accessions might have different N-source assimilation preferences which might be reflected in differential expression and responsiveness of N-related genes.

## Results

### Accession differences in biomass, NUE, NUpE and NUtE, grown at stationary N supply

*Arabidopsis thaliana* plants of the accessions Columbia-0 (Col-0), Shakdara (Sha), Edinburgh (Edi-0), Burren (Bur-0) and Tsushima (Tsu-0) represent contrasting accessions with different nitrate uptake- and use- efficiency^12^. These were grown hydroponically for 5 weeks with intermediate (0.4 mM) and high (8 mM) concentrations of inorganic nitrogen in form of ammonium nitrate. These concentrations are comparable to the total nitrogen concentrations used previously^12^. An important difference to previous studies and to natural conditions was that all plants had unlimited access to N (but N was supplied at different concentration), as the N in the nutrient solution of the large pots was kept constant. Furthermore, the high nitrogen condition was repeated with a different ammonium/nitrate ratio, were nitrate (~8 mM) was supplied in 40× excess to ammonium (0.2 mM). These concentrations were chosen to reflect the lower ammonium availability and mobility typically found in soils.

**Figure 1:**
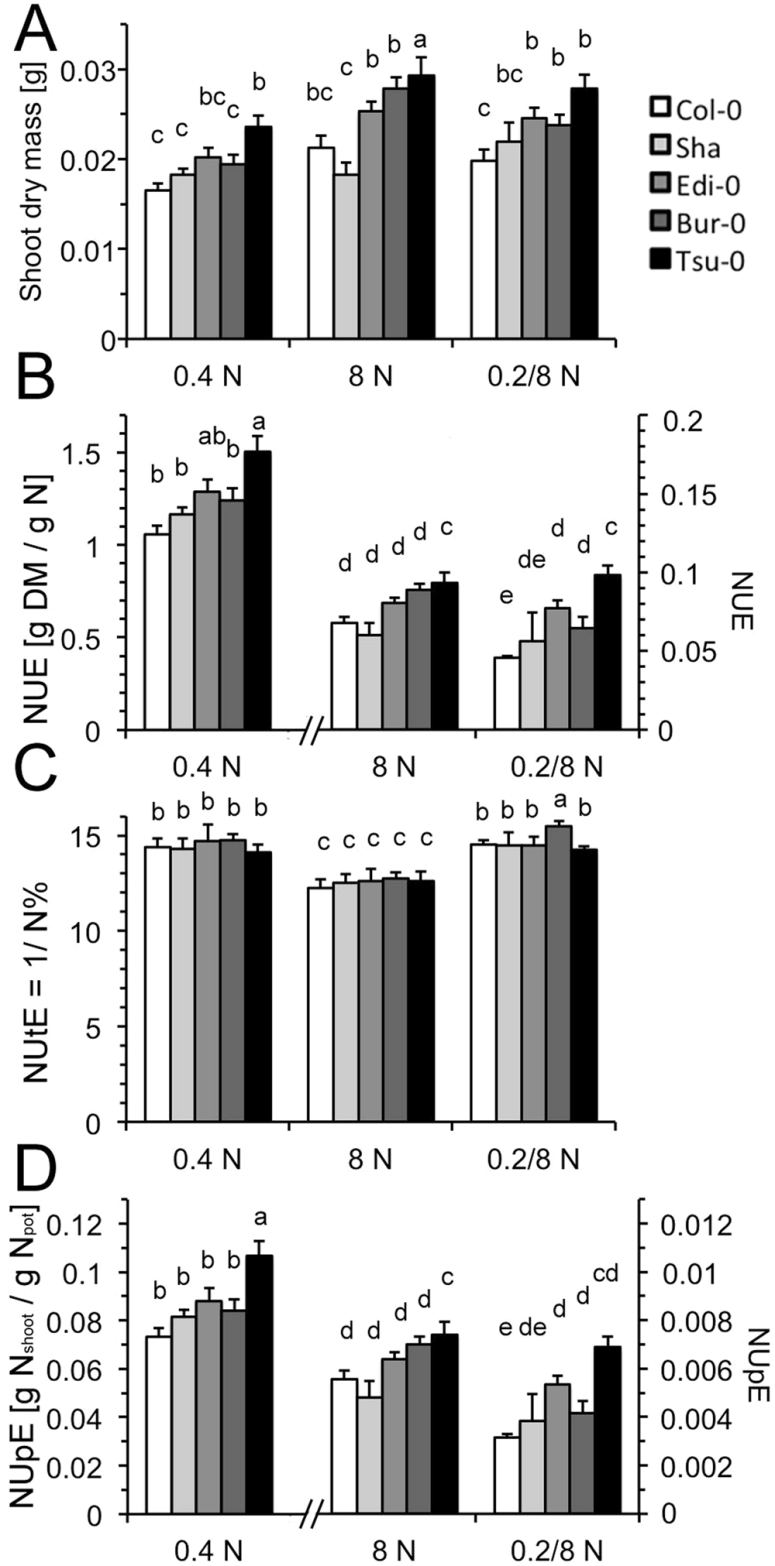
Nitrogen Use Efficiency parameters of contrasting *Arabidopsis* accessions. Plants were grown for 5 weeks on the indicated nitrogen conditions. (**A**) Shoot biomass (**B**) NUE, (**C**) NUtE (=1/shoot N%) (**D**) NUpE. Note the different scales for 0.4 N (=0.2 mM NH_4_NO_3_), left axis, and 8 N (=4 mM NH_4_NO_3_) and 0.2/8 N (=0.1 mM (NH_4_)_2_SO_4_ + 8 mM KNO_3_), right axis. NUE = Nitrogen use efficiency (dry shoot biomass/N in pot), NUtE = Nitrogen utilization efficiency (dry shoot biomass/shoot N), NUpE = Nitrogen uptake efficiency (shoot N/N in pot). Three independent repetitions with each n = 6 per accession and N-regime were performed. Data are given as means ± SD from one experiment to avoid normalization between different experiments. Statistically significant differences (ANOVA, p < 0.01) are indicated with different letters.

High nitrogen supply did marginally increase the shoot biomass of all genotypes, with the biomass tending to increase with the following sequence: Col-0 < Sha < Edi-0 < Bur-0 < Tsu-0 (Fig. 1A), in agreement with the results found by Chardon *et al*.^12^. However, the differences among the accessions were only significant in a few cases. Higher biomass and lower N in the nutrient solution were associated with higher NUE, which was defined as shoot dry biomass/available N in the pot (Fig. 1B). Because the pots represented a large nutrient reservoir that was frequently replenished, the large N amount in the nutrient solution little changed throughout the experiment. Only relative NUE differences among the accessions for individual N treatments are therefore considered here. Both at the intermediate and nitrate-dominated high N supply, the accessions little differed in their N concentrations (N%). Their N utilization efficiency (NUtE), a measure of the efficiency of the conversion of shoot N into shoot biomass, was therefore similar, as it is the inverse of N% (Fig. 1C). With high ammonium in the nutrient solution (4 mM), higher N% and lower NUtE was found (Fig. 1C). In all three conditions, the root accounted only for 10–16% of the total dry biomass and was not significantly different among the accessions. Interestingly, the uptake efficiency (NUpE), representing the capture of (unlimited) nitrogen from the hydroponics solution paralleled the increase in the biomass among accessions. But again, NUpE was not always statistically significantly different between genotypes (Fig. 1D). Only the performance of Col-0 (least NUE and NUpE) and Tsu-0 (highest NUE and NUpE) robustly differed throughout the experiments.

### Accession performance with limited N amount

**Figure 2:**
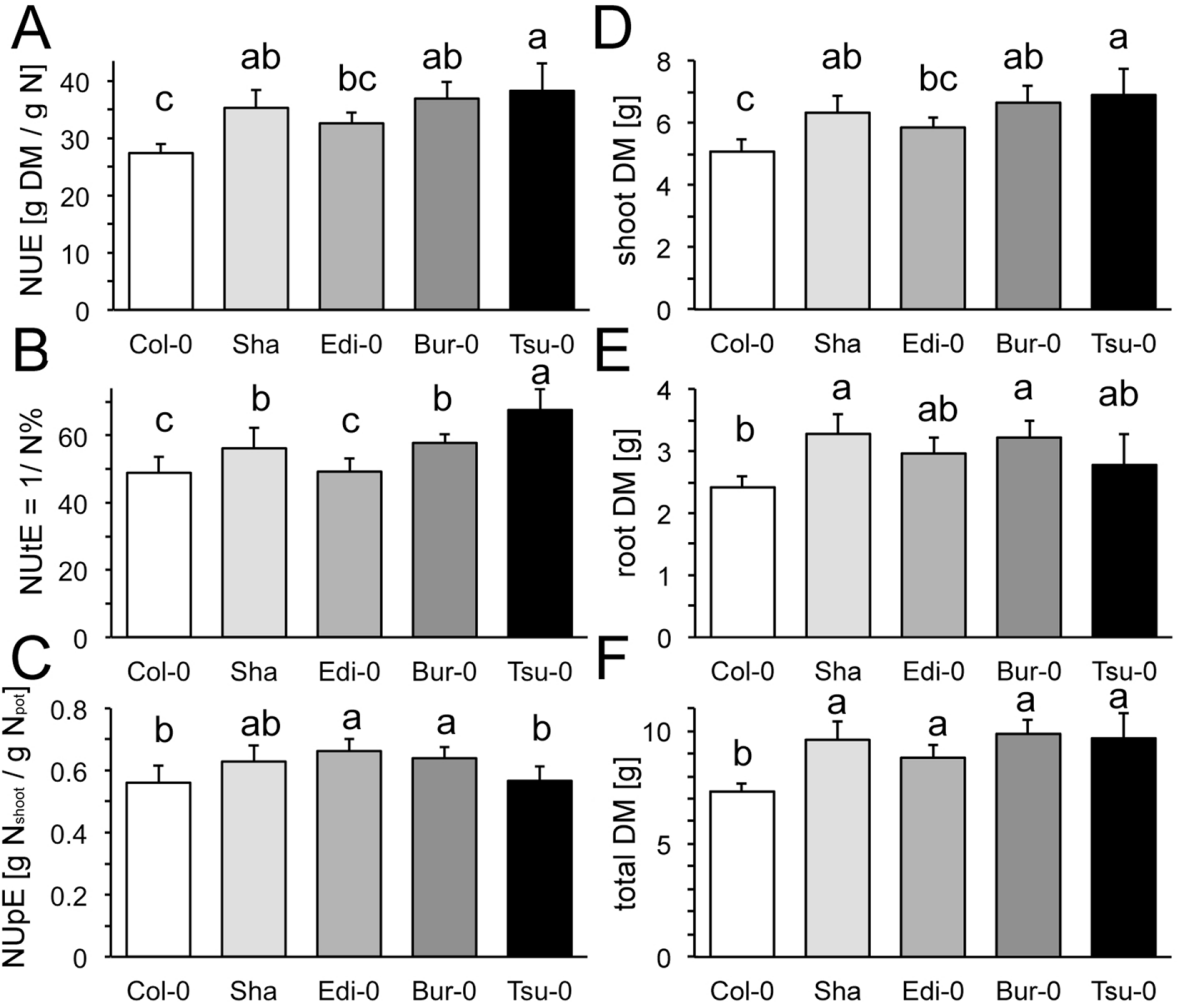
Nitrogen Use Efficiency parameters under N restriction. Individual plants were grown for 5 weeks per pot with 180 µg nitrogen as NH_4_NO_3_. (**A**) NUE, shoot dry biomass per 180 µg N in pot (**B**) NUtE (=1/shoot N%) (**C**) NUpE. NUE = Nitrogen use efficiency (dry shoot biomass/180 µg N in pot), NUtE = Nitrogen utilization efficiency (dry shoot biomass/shoot N), NUpE = Nitrogen uptake efficiency (shoot N/180 µg N in pot). Shoot (**D**), root (**E**) and total (**F**) dry mass. Data is given as means ± SD from a single experiment to avoid normalization. Different letters indicate significant differences with p ≤ 0.01 (ANOVA).

The high shoot N concentrations even at intermediate N indicated that the plants did not suffer from N deficiency, which occurs at below ~0.2 mM N. However, at very low N concentration in the nutrient solution, the root N uptake will quickly and significantly change the N concentration in the external nutrient media. Furthermore, plants in their natural environment rarely have unlimited, continuous steady access to very low N. We therefore considered a situation more meaningful in which a restricted amount of N (180 µg/plant) was applied once and individuals were allowed to utilize this amount to build up biomass during their entire growth period. Under these conditions, all accessions allocated between 41% (Tsu-0) and 52% (Sha) into their root biomass and showed visible chlorosis in the leaves. The shoot biomass and NUE (which is proportional to the shoot dry mass, as all plants obtained the same amount of ammonium nitrate) increased in the following sequence: Col-0 < Edi-0 < Sha < Bur-0 < Tsu-0 (Fig. 2A). This order is highly similar to the one obtained in the other experiments at constant higher N supply, suggesting the genotype differences in NUE are robust, irrespective of how N is applied and whether ammonium is supplied. Interestingly, under these conditions the accessions differed in their N concentrations (N%) and in their NUtE, which is in contrast to the situation at sufficient and constant N supply. The latter represents the shoot biomass per shoot N, with Col-0 and Tsu-0 again on opposite ends (Fig. 2B). Interestingly, however, Col-0 and Tsu-0 appeared to have similar NUpE that was slightly lower than the NUpE from the other accessions, suggesting that with restricted N, their capture of N via the roots was lower (Fig. 2C). The dry mass of the total plants, shoots and roots is also given and indicated that Edi-0, Sha, Bur-0 and Tsu-0 were quite similar (Fig. 2D–F).

### Labeling of ammonium- and nitrate reveals preference for nitrate in Tsu-0

**Figure 3:**
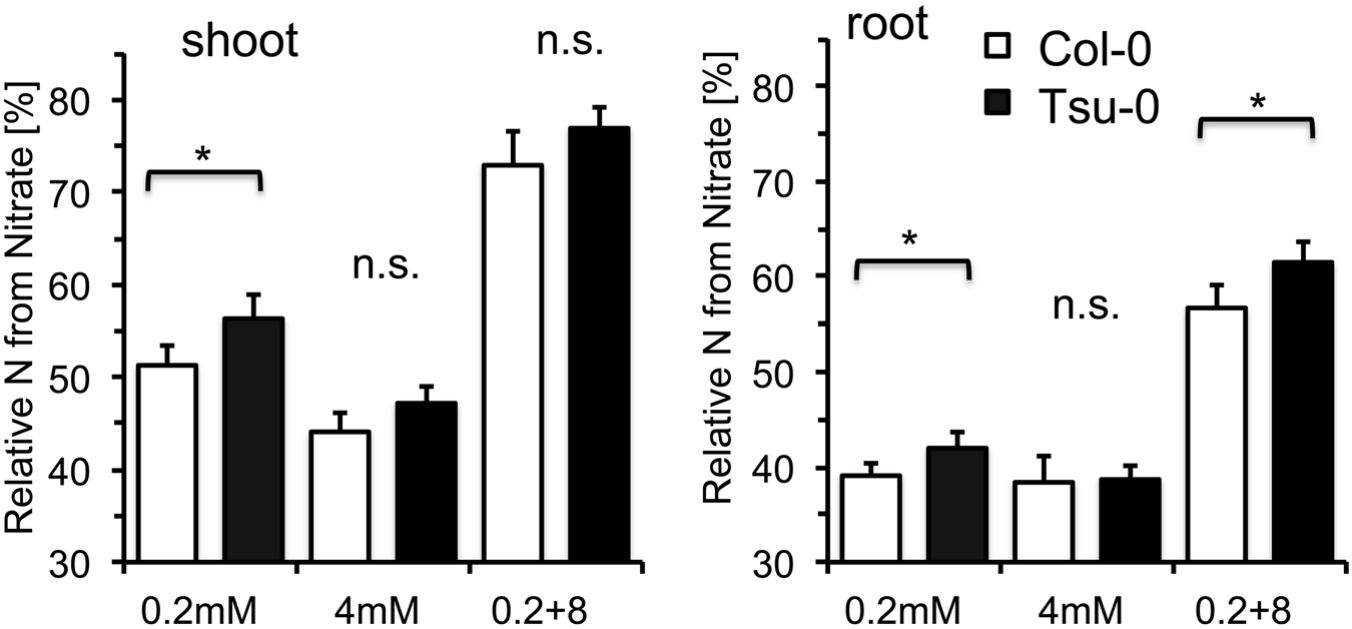
Preferential uptake of different N-forms in *Arabidopsis* accessions Col-0 (white bars) and Tsu-0 (black bars). Relative ^15^N isotope found in shoots (left) and roots (right) grown on mixed nitrogen sources (a fraction of either NH_4_^+^ or NO_3_^−^ was ^15^N labeled). Data are given in % of total N derived from nitrate. N supply as: 0.2 mM NH_4_NO_3_ (left), 4 mM NH_4_NO_3_ (middle) and 0.2 mM NH_4_^+^ + 8 mM NO_3_^−^ (right). Statistically significant differences (ANOVA, p < 0.05) are indicated by an asterisk.

For further analysis, we focused on the two most distinct, consistently significantly different accessions, Col-0 und Tsu-0. In nitrate-dominated nutrition, the differences in NUE of Tsu-0 and Col-0 were largest (Fig. 1B), suggesting that these accessions might differ in their usage ratios of ammonium and nitrate. We therefore isotopically labeled a defined fraction (10%) of either ammonium or nitrate and quantified the relative amount of ^15^N label in the final biomass. The nitrogen assimilation pathways in plants weakly discriminate against the heavier ^15^N isotope, leading to a change (fractionation) in the isotopic composition in the ‰ range of plant tissues relative to the natural nitrogen isotope abundance ratio (0.37% ^15^N)^17^. However, with massive labeling of one nitrogen source (10% as ^15^N), this fractionation effect becomes negligible relative to the isotope amount acquired by one specific N-form. After continuous 5-week supply to hydroponic cultures, the abundance of the ^15^N label in the total N was quantified. The majority of this ^15^N label then represents assimilated N from the N source that was ^15^N labeled, e.g. ^15^NH_4_^+^, while only a very minor fraction of this label corresponded to ^15^NH_4_^+^ from its natural abundance in all N (0.37% ^15^N). As controls, we also quantified the fraction of N that was taken up and that was assimilated with reverse labeling (e.g. ^15^NO_3_^−^). Furthermore, we confirmed that in samples in which both nitrogen forms were ^15^N labeled, approximately all plant N was represented by ^15^N. The fraction of nitrogen in Col-0 and Tsu-0 that was derived from ammonium was consistently higher in the root than in the shoot (Fig. 3). Differences between accessions were only significant at the lower N concentration and with nitrate-dominated high N supply in roots.

In Col-0, roughly half of the nitrogen acquired in the shoot originated from nitrate, while this fraction was only ~40% in roots. A slightly higher share was measured for Tsu-0, which also at higher N tended to use more N from nitrate, compared to Col-0 (Fig. 3). Strong accumulation of ^15^N-label in the shoot was expected when ^15^NO_3_^−^ was supplied at 40-fold higher concentration than NH_4_^+^, but even then, around 40% of N in the roots and around 25% in the shoots were derived from ammonium. The differences between the accessions were lost with 4 mM ammonium, where unspecific low affinity ammonium influx, which is little regulated, dominated the N uptake^18,19^. Massive uncontrolled influx of ammonium (Fig. 3) may then obscure the selective uptake via accession-specific uptake and root to shoot transfer systems.

### Superior growth of Tsu-0 on nitrate as sole nitrogen form

**Figure 4:**
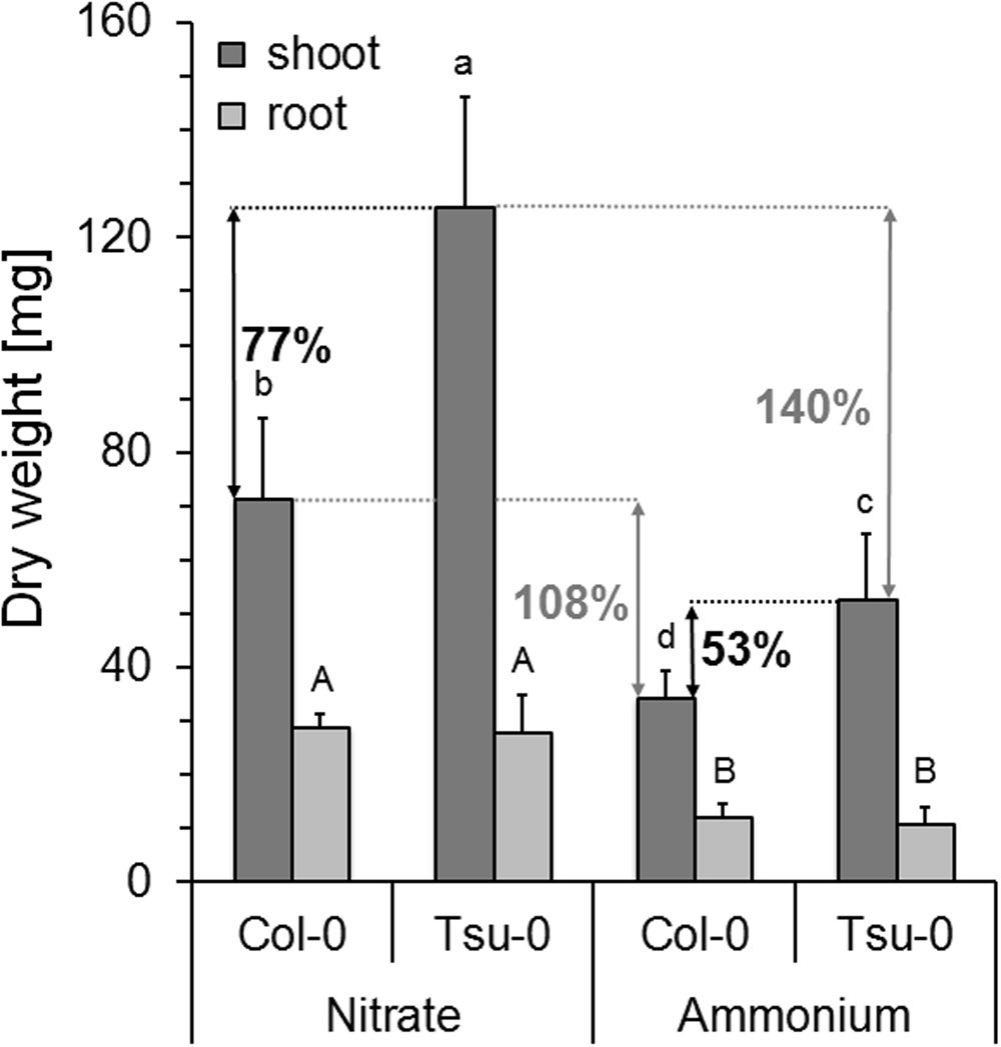
Superior growth of Tsu-0 grown under exclusive nitrate supply. Shoot and root dry weight [mg] of Col-0 and Tsu-0 plants grown in ammonium nitrate for 14 days and then transferred to single nitrogen sources for 21 days. Data represent a total of n = 21 plants per accession and N-regime. Data is given as means ± SD. The symbols A and B indicate significant differences (ANOVA) in root dry weight (p < 0.01), a, b, c, d indicate significant differences in shoot dry weight (p < 0.01). Black numbers [%] indicate growth increase of Tsu-0 compared to Col-0 in the respective N-form. Gray numbers indicate the growth increase of the respective accession between growth in nitrate, compared to growth in ammonium.

To further quantify whether the superior growth of Tsu-0 specifically depended on nitrate, we compared Tsu-0 and Col-0 plants grown in ammonium or nitrate as sole N form. We addressed growth differences of these two accessions by shoot and root dry weight. To prevent plants from ammonium toxicity, the N concentration was reduced, the nutrient media were pH buffered and seedlings were initially grown for 14 days (when they are most sensitive) in 1.5 mM ammonium nitrate, for equal establishment. Only then, plants were exposed to either ammonium or nitrate as the sole N-source (3 mM total N). Plant growth performance (root and shoot dry weight) was then separately analyzed after further 21 days. These experiments confirmed the higher biomass production of Tsu-0 (see Figs 1 and 2) in both N-forms. As expected, Tsu-0 showed superior growth stimulation by nitrate (Fig. 4). The shoot biomass of Tsu-0 was increased by 77%, compared to the Col-0 plants. Less difference was visible in ammonium-grown plants, where the Tsu-0 shoot biomass exceeded Col-0 only by 53% (Fig. 4). With nitrate, Col-0 showed an increase in shoot biomass by 108% compared to ammonium, while the biomass increase in Tsu-0 was 140%. The root biomass of both accessions did not significantly differ in both N-conditions.

### Ammonium and nitrate-specific gene expression in Col-0 and Tsu-0 roots

Taking into account that apparently the NUpE was the driver for different NUE, we took a closer look at differential gene expression in the roots. We addressed this by microarrays with mRNA extracted from ammonium- or nitrate-adapted plants and validated these data by qPCR. To minimize for developmental differences and differentiate between nitrate and ammonium-specific gene expression, we grew Tsu-0 plants initially with ammonium nitrate and only for the last 5 days before mRNA extraction changed to unique N-forms. This was done in parallel with Col-0, of which the data were previously published^20^.

**Figure 5:**
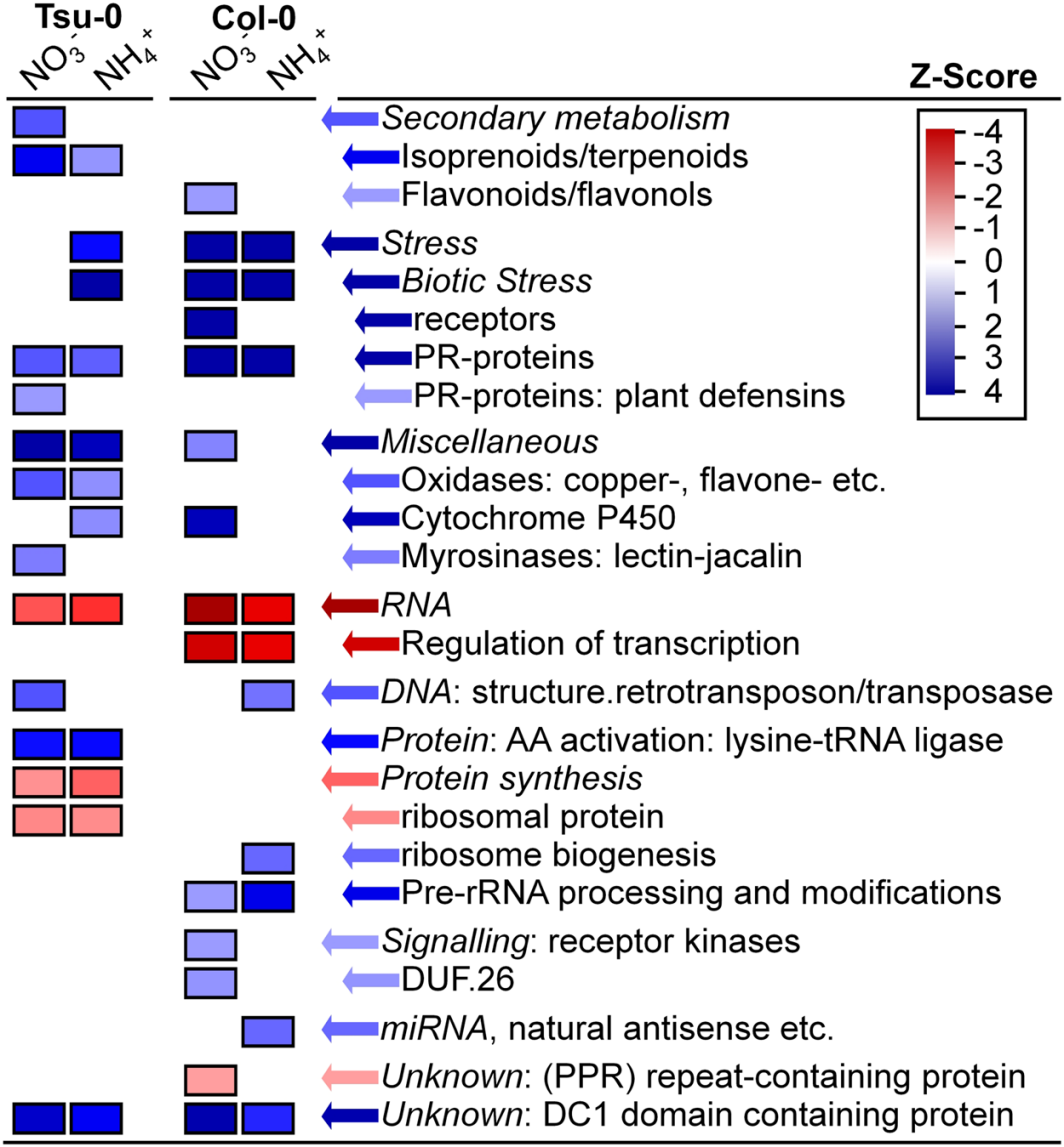
Overrepresentation analysis of Col-0 and Tsu-0 N-form-specific root transcriptomes. Overrepresented MapMan bins of 35 days old Col-0 or Tsu-0 plants grown on nitrate or ammonium as sole nitrogen source for five days. Comparisons were taken between both accessions grown with the same N-form in the nutrient solution (e.g. Col-0 NH_4_^+^ vs. Tsu-0 NH_4_^+^). Conducted with Fisher’s exact test and Benjamini-Hochberg corrected p-values, which were Z transformed (e.g. p = 0.05 ≜ Z = 1.96).

Using PageMan, overrepresented MapMan bins were identified, which represent differentially expressed functional gene categories between the two accessions (Fig. 5). As expected, notable differences in gene expression between the accessions were identified, but gene categories related to nitrate assimilation, nitrogen-uptake, -translocation and -metabolism were not among the differentially over-represented categories. In many cases, differentially expressed gene categories were similar between the two N-forms, but differed between the accessions. For example, stress related genes were overrepresented in Col-0, while secondary metabolism related genes were overrepresented in Tsu-0 (Fig. 5).

**Figure 6:**
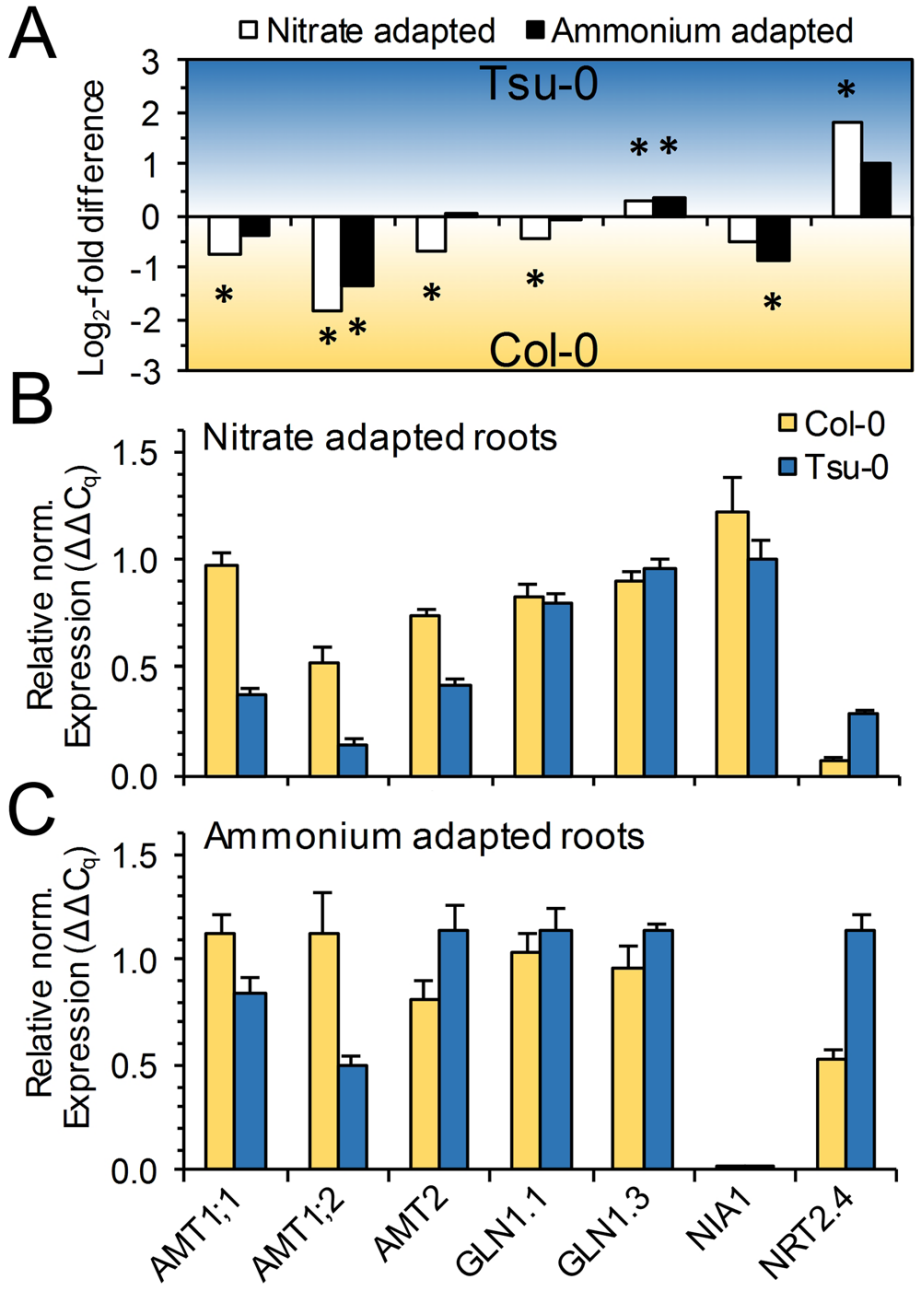
N-related genes which were significantly differentially expressed between Tsu-0 and Col-0 and their validation by qRT-PCR. (**A**) Significantly differentially expressed N-related genes derived from the transcriptome analysis. Negative Log_2_-fold values indicate a higher expression in Col-0 while positive Log_2_-fold values indicate higher expression in Tsu-0. White bars show the comparison of nitrate adapted, black the comparison of ammonium adapted plants. (**B**,**C**) Show qPCR verification of the differential expression of N-related genes in nitrate-adapted plants (**B**) and ammonium-adapted plants (**C**).

While PageMan overrepresentation analysis revealed no nitrogen uptake and -assimilation relevant functional gene classes, we focused on N-nutrition specific MapMan-Bins 12.1 (N.metabolism), 12.2.2 (ammonia.metabolism), 34.4 (transport.ammonium), 34.5 (transport.nitrate) and screened these for significantly (FDR < 0.05) differentially expressed genes between Tsu-0 and Col-0. The expression data confirmed a generally higher expression of AMT genes in Col-0, which were even increased in nitrate-grown plants (Fig. 6). Nitrate preferences of Tsu-0 might be caused by the higher expression of the high affinity nitrate transporter NRT2;4. The differential expression of some N-related genes was mostly verified by qPCR (Fig. 6B,C), but the minor differences in nitrogen assimilation genes were not confirmed by qPCR. Overall, the data are consistent with a higher ammonium usage by Col-0 than by Tsu-0 and a higher nitrate usage by Tsu-0 compared to Col-0. The higher nitrogen use efficiency of Tsu-0, however, is poorly explained by these data.

### Nitrate-depletion responses are similar in Col-0 and Tsu-0

**Figure 7:**
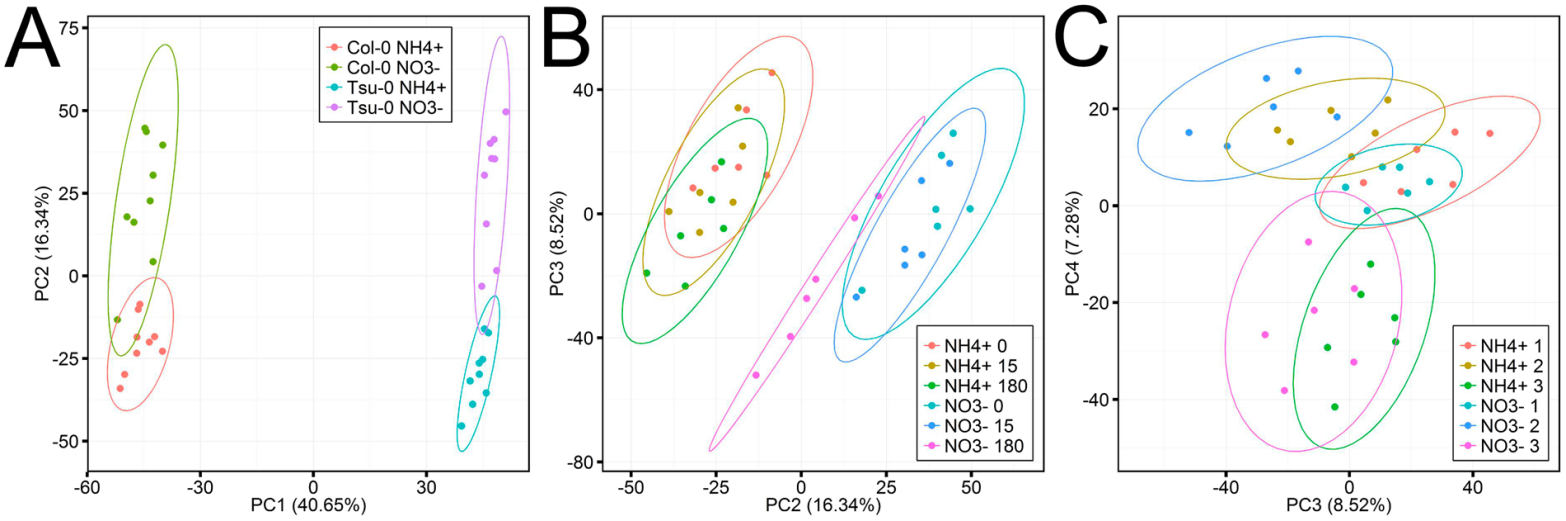
Principal Component Analysis of microarray data of all N-depletion experiments conducted with the accessions Col-0 and Tsu-0. Left: PC1 vs. PC2, data points are grouped for accession and pre-treatment, center: PC2 vs. PC3, data grouped after pre-treatment and time-point, right: PC3 vs. PC4 grouped for replication and pre-treatment.

We therefore considered the possibility that the higher NUpE and NUE in Tsu-0 might result from improved perception, signaling and/or gene expression response to the lack of external nitrogen (especially of nitrate). This was addressed by a depletion experiment, in which Tsu-0 was expected to react with higher transcriptional plasticity to the nitrogen deprivation. In this experimental setup plants were deprived of ammonium or nitrate for 15 min and 180 min^20^. A principal component analysis (PCA) with normalized microarray intensity-data derived from Tsu-0 and Col-0, adapted to nitrate or ammonium, respectively, and then deprived of nitrogen was conducted (Fig. 7). Surprisingly, PC1 that explained 40.7% of the experimental variance clearly separated the datasets into two groups representing general accession differences. The different nitrogen forms were reflected by PC2, which still accounted for 16.3% of the variance. Principal component 3 and those of higher magnitudes separated the data according to the time-course of N-depletion and the three replications of the experiment.

**Figure 8:**
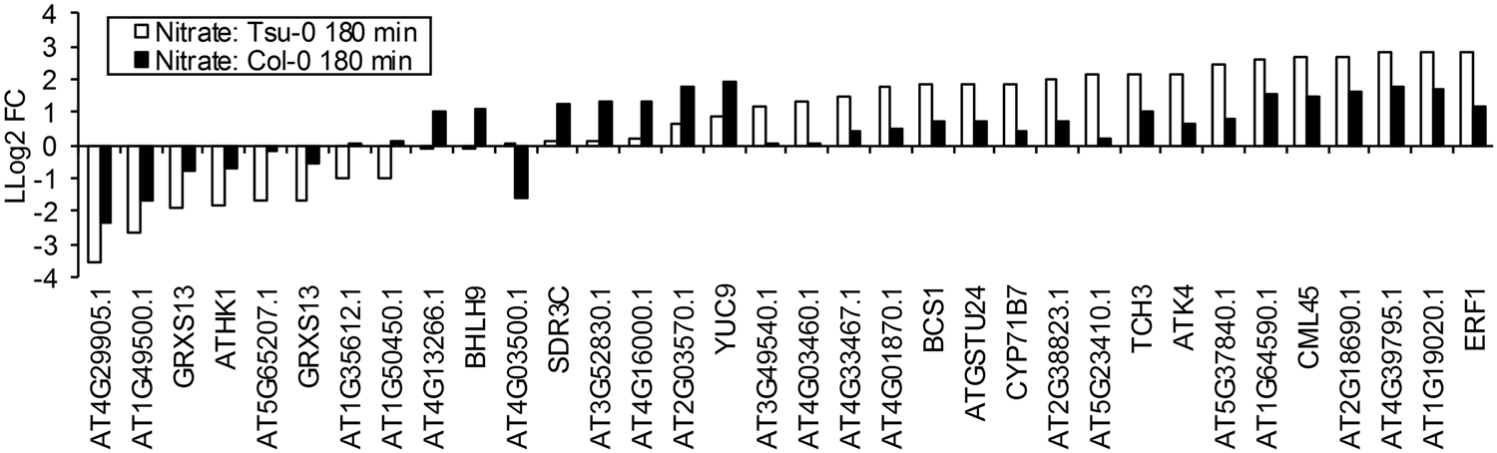
Accession-specific differential gene expression after 3 h nitrate depletion. Log_2_ fold-changes for the genes significantly differentially regulated between the accessions were calculated between the sample 3 h after onset of N-depletion.

Surprisingly, the general response to N-depletion in nitrate-adapted plants was highly similar in Col-0 and Tsu-0 plants. Transcripts of *SAUR6* (*AT2G21210*), *LBD37* (*AT5G67420*), *LBD39* (*AT4G37540*) and *HHO1* (*AT3G25790*) were among the genes which earliest responded to nitrate depletion. These were down-regulated after nitrate deprivation. A common down-regulation of these and other major nitrate-metabolism genes was observed after 3 hours^20^. In a direct comparison of the depletion responses of both accessions, not a single significantly different expression change of any N-related gene was detected. However, a few genes responded towards N-depletion with slightly delayed or opposite regulation in one of the two accessions. As these genes were mostly unknown, they could not be related to the known nitrate assimilation network, but these may still reflect a differential nitrate-depletion response among the accessions and may be worth further study (Fig. 8). In Col-0, the ammonium-adapted roots lacked a rapid, meaningful, detectable response towards ammonium-depletion. Tsu-0 also did not respond with expression changes to the depletion of ammonium within 3 h.

## Discussion

*Arabidopsis* accessions collected from different geographic sites differ in their responses to environmental conditions. Especially the induction of flowering as a response to ambient temperature, is highly variable between accessions. Differences among accessions were, however, also described for their uptake of nutrients and the efficiency of translating this uptake into yield. Detailed investigations of the nitrogen use efficiency in *Arabidopsis* accessions in sand culture that was fertigated with nitrate solutions revealed a surprisingly large variance among genotypes. Among a core collection of accessions, 5 NUE classes emerged from these experiments with Col-0 and Tsu-0 at opposite ends^12^. In these growth conditions, poor NUE performers, such as Sha and Col-0, were characterized by high N% and high NUpE, irrespective of the N supply. Edi-0 was intermediate and Bur-0 more similar to Tsu-0, which had the lowest NUpE and N% under low and high N, but the highest NUE^12^. Interestingly, the same NUE sequence of accessions emerged for most of our experiments, in which nitrate was replaced by mixed ammonium nitrate solutions and hydroponics, with restricted or unlimited N supply, were used. This indicated that NUE genotype comparisons were robust and quite insensitive to the study system, nitrogen form and external concentration. Higher biomass was always associated with higher overall NUE (Figs 1A,B, 2). However, the NUpE, reflecting the N capture by the roots, drastically changed and was even reversed in the different systems. Both at the intermediate and high N supply in hydroponics, the accessions differed little in their N concentrations (N%). They therefore showed similar N utilization efficiency (NUtE) at a given N-supply, but increasing NUpE from Col-0 to Tsu-0, which is in contrast to the nitrate fertigated sand culture (Fig. 1C,D)^12^. That the nutrient uptake and NUpE is crucial to improve NUE is supported by several examples in the rice crop, where increased expression or the introduction of novel nitrate transporters improved NUE^8,9^. Our study confirmed that the accession Col-0, commonly used as reference genotype, possesses a very low NUE with weak influence of the N-supply on the biomass. Other accessions, Sha, Edi-0 and Bur-0, were intermediate (Figs 1, 2). The accessions differed little in their high N-concentration of approximately 6–8% in the shoots, with intermediate and high N supply, respectively.

Furthermore, our results identified different nitrogen source preferences of Col-0 and Tsu-0, when ammonium and nitrate were applied simultaneously or individually (Figs 3, 4). Generally, ammonium appeared to be the preferred N source in roots. Furthermore, despite a 40-fold excess of nitrate over ammonium, a substantial amount of N in roots (around 40%) and shoots (around 25%) was derived from ammonium. Interestingly, the largest differences in NUE and growth between Col-0 and Tsu-0 were observed at low N supply (0.2 mM NH_4_NO_3_) and in the 8 mM nitrate/ 0.2 mM NH_4_^+^ condition, but not with 4 mM NH_4_NO_3_. This reflects the preference of the uptake, assimilation and potentially translocation of nitrate in Tsu-0 with 0.2 mM NH_4_NO_3_ and 8 mM nitrate + 0.2 mM NH_4_^+^.

Despite the differences in the biomass and NUpE in Col-0 and Tsu-0, the root transcriptome failed to specifically identify nitrogen or nitrate uptake and assimilation categories as significantly differentially regulated between these genotypes (Fig. 5). However, a few differences in the expression of nitrogen transporter and assimilation genes were identified. Their differential expression was partially confirmed by RT-qPCR (Fig. 6). Tsu-0 incorporated significantly higher amounts of ^15^N from nitrate compared to Col-0 (Fig. 3). If nitrate was supplied as sole nitrogen source, the shoot dry mass of Tsu-0 increased much larger than that of Col-0 (Fig. 4). A significant difference in the transcriptomes was found for other functional gene categories, unrelated to nitrogen nutrition, but not in N-related categories (Figs 5, 7). These transcriptome differences were found in categories that might influence biomass by other means. Indeed, biomass was most closely correlated with NUE and is a complex trait influenced by thousands of genes. It is therefore challenging to decipher single contributing genes and their function. In fact, functional gene classes overrepresented in Tsu-0 or Col-0, respectively, include secondary metabolism-related genes involved in isoprenoid/terpenoid synthesis (over-represented in Tsu-0). Furthermore, stress-related genes were over-represented in Col-0 (Fig. 5). How these functional gene classes affect NUE remains speculative. The high-affinity nitrate transporter gene *NRT2*.*4*^21^ was higher expressed in nitrate adapted roots of Tsu-0 and may have an influence on its higher nitrate preference. By contrast, in Col-0, several high-affinity ammonium transporter genes (*AMT1;1*, *AMT1;2* and *AMT2*) were higher expressed in nitrate and ammonium (Fig. 6). Accession-specific differential expression of several nitrate transporters and assimilation genes was observed before in the accessions Col-0, Ga-0, Sha and Ws-0^15^.

In agreement with the observation that the type of N-supply (unlimited at certain level vs. decreasing amount) little influenced the NUE accession sequence. The N-depletion response was highly similar in Tsu-0 and Col-0. Only after 180 min, a few differences in mostly unknown (or unspecific) genes were detected (Figs 7, 8). These might result from the possibility that 3 h was a too short duration to detect clear differences between Tsu-0 and Col-0 in their adaptation towards nitrogen depletion. Before transcriptional differences occur, remobilization of nitrogen from stores is expected, which may not require major gene expression differences. The N-depletion response therefore did not identify known genes that might be correlated with NUE, NUpE or the preference of nitrate by Tsu-0. However, a metabolite time course analysis of the deprivation response may be valuable in the future to test for other genotype differences.

We conclude that NUE and NUpE differences between *Arabidopsis* accessions are not necessarily manifested in differences in expression of N-associated genes in roots. Therefore, manipulating the expression or function of single nitrogen related genes at least in *Arabidopsis* might not be sufficient to address the important issue of improving NUE. Nevertheless, robust preferences for single nitrogen sources exist and may partially be explained by steady state differences in some nitrogen uptake and assimilation genes. Although different accessions have different preferences for assimilating ammonium or nitrate, robust differences in the nitrogen use efficiency between *Arabidopsis* accessions are independent of whether plants were fed with nitrate or ammonium nitrate.

## Materials and Methods

### Hydroponic plant culture of different accessions

Imbibed and stratified (4 °C for 3 days), seeds of different *Arabidopsis thaliana* accessions (which were only collected from distinct ecosystems but then poropagated similar and are therefore not called ecotypes): Columbia-0 (Col-0), Shakdara (Sha), Edinburgh-0 (Edi-0), Burren-0 (Bur-0) and Tsushima-0 (Tsu-0) were grown in aerated hydroponic culture^22^, with minor modifications, under short day conditions in a growth chamber (10 h daytime with light intensity of 200 µmol m^−2^ sec^−1^, 22 °C and 14 h night, 18 °C). Modified ¼ Hoagland’s nutrient solution (1 mM KH_2_PO_4_; 0.5 mM MgSO_4_; 0.1 mM Na_2_EDTA-Fe; 1 mM CaCl_2_; 9 μM MnSO_4_; 0.765 μM ZnSO_4_; 0.32 μM CuSO_4_; 0.016 μM Na_2_MoO_4_; 46 μM H_3_BO_3_, pH 6 adjusted with KOH) was used. Nitrogen was given in balanced amounts of ammonium and nitrate in form of 0.2 mM NH_4_NO_3_, 4 mM NH_4_NO_3_ and in form of a dissimilar ammonium and nitrate treatment with 0.1 mM (NH_4_)_2_SO_4_ and 8 mM KNO_3_. Nutrient solution was replaced weekly during the first two weeks and subsequently every four days until plants were 35 days old. Single plants were harvested, washed in 1 mM CaSO_4_, separated into roots and shoots and weighed out for fresh weight. Afterwards, plant parts were frozen in liquid N_2_. Dry weight was determined from lyophilized plant material. For individual conditions at least 6 plants were analyzed (see figure legends for details) and shoot fresh and dry weight, root fresh and dry weight, shoot N%, root N%, shoot N, root N, total fresh and dry weight, N%, total N and root/shoot ratio were determined.

### Definition of NUE, NUtE, NUpE

NUE (Nitrogen use efficiency) was calculated as dry shoot biomass/N in pot, NUtE (Nitrogen utilization efficiency) as dry shoot biomass/shoot N and NUpE (Nitrogen uptake efficiency) as shoot N/N in pot. Note that NUtE = 1/shoot N% and NUE = NUtE × NUpE.

### Determination of uptake of preferential N-forms

In order to analyze accession-specific N-form preferences, plants were grown in hydroponic culture with the same N-concentrations (0.2 mM; 4 mM; 0.2 mM NH_4_^+^  + 8 mM NO_3_^−^) as mentioned above. However, one or both nitrogen-forms were labeled with ^15^N (^15^NH_4_^15^NO_3_; ^15^NH_4_NO_3_; NH_4_^15^NO_3_) and (^15^NH_4_)_2_SO_4_ or K^15^NO_3_ (each with 10 atom% ^15^N). Reverse labeling was used as control. Total N (N%) and ^15^N-concentration were determined separately for root and shoot from ~1 mg ground, lyophilized plant material by isotope ratio mass spectrometry (Thermo-Fisher Scientific). The ^15^N-fraction was consequently equivalent to the uptake and assimilation of the labeled nitrogen form over the 35 days of plant growth.

### Plant culture and N-depletion experiment for Microarray Analysis

Plants of Tsushima-0 (Tsu-0) accession of *Arabidopsis thaliana* were grown in hydroponic culture in a growth chamber with short day conditions, similarly as described above. Modified ¼ Hoagland nutrient solution adjusted with KOH to pH 6 was used. Nitrogen was given in form of 1.5 mM NH_4_NO_3_, 1.5 mM (NH_4_)_2_SO4 or 3 mM KNO_3_, respectively. Three experimental independent replicates were used for each condition. The first 10 days after germination nutrient solution was replaced and the following 25 days every week during the total growth period of 35 days. N-depletion was conducted identically as described elsewhere^20^. Nitrogen was replaced by 1.5 mM K_2_SO_4_ in the N-depleted nutrient solution.

### RNA-Extraction

Total RNA was extracted from 100 mg aliquots of frozen root material with innuPREP Plant RNA Kit (Analytik Jena, Germany) with an additional in-column DNAse digestion-step (RNase-Free DNAse Set, QIAGEN).

### Microarray analysis

Total RNA samples of each replicate were analyzed by Oaklabs GmbH (Henningsdorf, Germany) on experimentally validated custom microarrays (ArrayXS Arabidopsis) based on Agilent 8 × 60 K microarray-chips (Agilent Technologies, Inc., Santa Clara, USA). Quality tests, dye-binding, hybridization and microarray scanning were conducted by Oaklabs. Agilent raw-intensity data were analyzed with the limma-package of Bioconductor^23^. Data were background-corrected, quantile-normalized between the arrays and probes with intensities less than 10% brighter than negative-control probes of each array were filtered. Approximately 23,400 transcripts remained detectable after filtering. The linear model analysis of limma was then used to identify differentially expressed genes between the samples. Depending on treatment, differentially expressed genes were defined by a log_2_ fold-change of ±1 or ±2 and a Benjamini-Hochberg corrected false discovery rate (FDR) lower than 0.05.

### Principal Components Analysis

PCA was conducted with normalized intensity-data of all microarray experiments of Col-0 and Tsu-0 accessions except control and 5 day-datasets in R with prcomp function and ggord plugin for ggplot2 graphics library.

### Overrepresentation Analysis

PageMan application of MapMan version 3.5.1^24^ was used to test for over-represented transcripts by use of Fisher’s exact test and Benjamini-Hochberg corrected p-values and gene-expression lists derived from limma.

### cDNA synthesis and quantitative RT-PCR

Of each sample 1 µg of total RNA was transcribed into cDNA with QuantiTect Reverse Transcription kit (QIAGEN GmbH, Hilden, Germany) following the instructions in the manual. For qRT-PCR cDNA was 10× diluted. 5 µl cDNA-dilution resembling 25 ng total RNA were used per reaction. Each reaction with 7.5 µl 2× KAPA SYBR Fast qPCR Supermix, 200 nM gene specific primer in a total reaction volume of 15 µl was conducted in technical triplicates. A two-step thermal cycling protocol with 40 cycles of 3 s 95 °C and 20 s at 60 °C after initial heating for 3 min at 95 °C was used. To confirm single amplicons, melt curves were recorded after final cycle heating from 65 °C to 95 °C with an increment of 0.5 °C per step. PCR-reactions were analyzed with the CFX384 Realtime-PCR Detection System/C1000 Thermal Cycler set (BioRad, Hercules, USA). Data analysis was conducted with CFX Manager Software v3.1 according to MIQE standards. The relative gene expression values (using the ∆∆C_q_ method) were normalized towards the expression of the reference genes SAND (At2g28390) and PDF2 (At1g13320)^25^. Averaged values were shown for three biological replicates of each condition with each. Primers used for qRT-PCR: *AMT1;1* (*At4g13510*): Fw: TATGGGCGGTGGAGGAAAAC Rv: CTCGGACGATATCCGCAACA, *AMT1;2* (*At1g64780*): Fw: CGACTCCTACACCGACCTTG Rv: TTTGGTGCCCGAACTCTTGT, *AMT2* (*At2g38290*): Fw: AAGGGACAAGCAAAGATCCCA Rv: ATCCCGCCACAAGTATCGTC, *GLN1*.*1* (*At5g37600*): Fw: TGTGAAGTGGCCTGTTGGTT Rv: TGTCTGCTCCAATACCGCAA, *GLN1*.*3* (*At3g17820*): Fw: AGCGTCGTCTCACTGGAAAG Rv: CACGTCCCACTCTCACTGAC, *NIA1* (*At1g77760*): Fw: GGCTACGCTTATTCTGGAGGAGGT Rv: TGGTGGTCAAGCTCACAAACACTC, *NRT2*.*4* (*At5g60770*): Fw: CTGGTGGAAACTTCGGCTCT Rv: CCATCCATGTCAGCCCTTGT. Reference Genes: *PDF2 (At1g13320)*^25^, Fw: TAACGTGGCCAAAATGATGC, Rv: GTTCTCCACAACCGCTTGGT, S*AND (At2g28390)*^25^, Fw: AACTCTATGCAGCATTTGATCCACT, Rv: TATTGCATATCTTTATCGCCATC.

### Statistical analysis

All data are given as mean + standard deviation, see details. One- or two- factorial ANOVA tests were applied to evaluate statistically significant differences. Accession x N-regime interactions were generally not significant. Significant differences are given as different letters or with a star (p < 0.05), same letters or n.s. indicate no significant difference.

### Data Availability

All data included in this manuscript will be made available online.
